# Detecting frontotemporal dementia syndromes using MRI biomarkers

**DOI:** 10.1016/j.nicl.2019.101711

**Published:** 2019-02-04

**Authors:** Marie Bruun, Juha Koikkalainen, Hanneke F.M. Rhodius-Meester, Marta Baroni, Le Gjerum, Mark van Gils, Hilkka Soininen, Anne M. Remes, Päivi Hartikainen, Gunhild Waldemar, Patrizia Mecocci, Frederik Barkhof, Yolande Pijnenburg, Wiesje M. van der Flier, Steen G. Hasselbalch, Jyrki Lötjönen, Kristian S. Frederiksen

**Affiliations:** a Danish Dementia Research Centre, Department of Neurology, Rigshospitalet, University of Copenhagen, Denmark; b Combinostics Ltd., Tampere, Finland; c Alzheimer Center Amsterdam, Department of Neurology, Amsterdam Neuroscience, Vrije Universiteit Amsterdam, Amsterdam UMC, Amsterdam, the Netherlands; d Institute of Gerontology and Geriatrics, University of Perugia, Perugia, Italy; e VTT Technical Research Center of Finland Ltd, Tampere, Finland; f Institute of Clinical Medicine, Neurology, University of Eastern Finland, Kuopio, Finland; g Neurocenter, neurology, Kuopio University Hospital, Kuopio, Finland; h Unit of Clinical Neuroscience, Neurology, University of Oulu, Oulu, Finland; i Medical Research Center, Oulu University Hospital, Oulu, Finland; j Department of Radiology and Nuclear Medicine, Amsterdam Neuroscience, Vrije Universiteit Amsterdam, Amsterdam UMC, Amsterdam, the Netherlands; k UCL institutes of Neurology and Healthcare Engineering, London, UK

**Keywords:** Dementia, Frontotemporal lobar degeneration, Differential diagnosis, Behavioral variant frontotemporal dementia, Primary progressive aphasia, MRI

## Abstract

**Background:**

Diagnosing frontotemporal dementia may be challenging. New methods for analysis of regional brain atrophy patterns on magnetic resonance imaging (MRI) could add to the diagnostic assessment. Therefore, we aimed to develop automated imaging biomarkers for differentiating frontotemporal dementia subtypes from other diagnostic groups, and from one another.

**Methods:**

In this retrospective multicenter cohort study, we included 1213 patients (age 67 ± 9, 48% females) from two memory clinic cohorts: 116 frontotemporal dementia, 341 Alzheimer's disease, 66 Dementia with Lewy bodies, 40 vascular dementia, 104 other dementias, 229 mild cognitive impairment, and 317 subjective cognitive decline. Three MRI atrophy biomarkers were derived from the normalized volumes of automatically segmented cortical regions: 1) the anterior vs. posterior index, 2) the asymmetry index, and 3) the temporal pole left index. We used the following performance metrics: area under the receiver operating characteristic curve (AUC), sensitivity, and specificity. To account for the low prevalence of frontotemporal dementia we pursued a high specificity of 95%. Cross-validation was used in assessing the performance. The generalizability was assessed in an independent cohort (*n* = 200).

**Results:**

The anterior vs. posterior index performed with an AUC of 83% for differentiation of frontotemporal dementia from all other diagnostic groups (Sensitivity = 59%, Specificity = 95%, positive likelihood ratio = 11.8, negative likelihood ratio = 0.4). The asymmetry index showed highest performance for separation of primary progressive aphasia and behavioral variant frontotemporal dementia (AUC = 85%, Sensitivity = 79%, Specificity = 92%, positive likelihood ratio = 9.9, negative likelihood ratio = 0.2), whereas the temporal pole left index was specific for detection of semantic variant primary progressive aphasia (AUC = 85%, Sensitivity = 82%, Specificity = 80%, positive likelihood ratio = 4.1, negative likelihood ratio = 0.2). The validation cohort provided corresponding results for the anterior vs. posterior index and temporal pole left index.

**Conclusion:**

This study presents three quantitative MRI biomarkers, which could provide additional information to the diagnostic assessment and assist clinicians in diagnosing frontotemporal dementia.

## Highlights

• Quantitative MRI biomarkers (API, ASI, and TPL) for detection of FTD and its subtypes.
• API differentiated FTD from other diagnostic groups with AUC of 83%.
• ASI and TPL showed highest performance for PPA subtypes.
• A subcortical bvFTD subtype resembling AD atrophy pattern seems undetectable for MRI.

## 1 Introduction

Frontotemporal dementia (FTD), the second most frequent early-onset neurodegenerative dementia disease, represents various clinical syndromes including behavioral variant frontotemporal dementia (bvFTD) and primary progressive aphasia (PPA) (Picard C., 2011; Rabinovici and Miller 2010). PPA may be further subdivided into subgroups including semantic variant PPA (svPPA) and non-fluent variant PPA (nfvPPA) (Gorno-Tempini et al. 2011). Determining these clinical FTD diagnoses can be challenging as the clinical symptoms and neuropsychological profiles overlap with other types of dementia, e.g., AD (Mendez et al. 2013; Ossenkoppele et al. 2015; Smits et al. 2012; Vijverberg et al. 2016). However, accurate and early diagnosis is important to ensure optimal counseling, care, and treatment.

Frontal and temporal lobe atrophy on magnetic resonance imaging (MRI), with relative preservation of posterior areas, represent the imaging hallmark of frontotemporal lobar degeneration (the neuropathological changes underlying FTD) (Neary et al. 1998). For bvFTD the areas with the most pronounced gray matter atrophy are typically the frontal lobes, the insula, and the anterior cingulate cortex (Pan et al. 2012; Schroeter et al. 2007; Whitwell et al. 2009). For nfvPPA the atrophy is predominantly left-sided in inferior-frontal and insular cortices, whereas for svPPA asymmetrical (commonly left-sided) anteroinferior temporal lobe and temporal gyrus atrophy is normally observed (Gorno-Tempini et al. 2004; Schroeter et al. 2007). Studies have demonstrated that visual rating of atrophy patterns provide useful diagnostic information based on simple and reliable scales (Harper et al. 2016). However, subtle atrophy in early stages of the disease and overlap in atrophy patterns between dementia types may reduce the utility of such approaches (Mendez et al. 2013; Mesulam et al. 2014). Moreover, visual image evaluation depends on the training and experience of the radiologist which may vary, especially outside specialized centers (Klöppel et al. 2008).

MRI provides a multitude of data for defining different imaging biomarkers, some of which require complicated post-processing and image analysis (Bouts et al. 2018; Möller et al. 2015b; Steketee et al. 2016a). Current imaging biomarkers available for detecting FTD have so far only obtained modest diagnostic performance (Canu et al. 2017; Harper et al. 2016, 2015; Meyer et al. 2017; Möller et al. 2015b). Accuracy may increase when combining different imaging biomarkers, but the combinations are often purely data-driven (Canu et al. 2017; Meyer et al. 2017; Möller et al. 2015a). From a clinical viewpoint such combinations might therefore not be intuitive or logical, whereas for clinicians it is preferable to operate with simple and easily understandable biomarkers. Furthermore, research studies focus predominately on differentiating bvFTD from AD or healthy controls, and are often performed in selected cohorts. Here, the relatively low prevalence of FTD and heterogeneity of a mixed memory population are seldom accounted for (Canu et al. 2017; Hogan et al. 2016; Meyer et al. 2017; Möller et al., 2015a, Möller et al., 2015b).

The main objective of this study was to develop and validate the diagnostic accuracy of simple automated MRI biomarkers for clinical diagnosis of FTD. We focused on the frontotemporal atrophy patterns for identification of an index for differentiation of FTD from all other dementia groups (non-FTD) (Neary et al. 1998). The asymmetrical atrophy patterns in nfvPPA and svPPA, and the anterior temporal lobe affection in svPPA formed the basis for the indexes developed for separation of PPA subtypes from non-FTD and bvFTD (Gorno-Tempini et al. 2004; Schroeter et al. 2007). First, we studied the ability of the biomarkers to differentiate FTD from non-FTD in a mixed memory clinic cohort. Second, we assessed the performance when differentiating FTD subtypes from one another.

## 2 Materials and methods

### 2.1 Participants

In this retrospective multicenter study, we included 1213 patients from two memory clinic cohorts. From the Amsterdam Dementia Cohort (ADC), which has been consecutively acquired at the Alzheimer center Amsterdam, Amsterdam UMC between 2004 and 2014, we included 614 patients (Van Der Flier et al. 2014; Van Der Flier and Scheltens 2018). From the PredictND multicenter cohort, which was based on consecutive sampling from 4 European centers, we included 599 patients (Bruun et al. 2019). This ADC + PredictND cohort consisted of patients with the following diagnoses: 341 AD, 66 dementia with Lewy bodies (DLB), 40 vascular dementia (VaD), 104 other dementias (e.g. Parkinson's disease with dementia, atypical parkinsonism, normal pressure hydrocephalus and dementia with uncertain etiology), 229 mild cognitive impairment (MCI), 317 subjective cognitive decline (SCD), and 116 FTD patients with the following FTD subtypes: 64 bvFTD, 30 svPPA, 8 nfvPPA, 10 right temporal variant FTD (rtvFTD), and 4 FTD with motor neuron disease (+MND). Based on purposive sampling an independent validation cohort of 200 patients was obtained from the Danish Dementia Research Centre (DDRC), Copenhagen University Hospital – Rigshospitalet, Denmark: 110 AD, 20 DLB, 28 VaD, 18 SCD, and 24 FTD with the following subtypes: 10 bvFTD, 12 svPPA, and 2 nfvPPA. Patients were eligible for inclusion if MRI of sufficient quality was available. For this purpose, visual inspection of scans was performed and scans containing large artifacts, very noisy images, and images with contrast agent were excluded.

All patients received a standardized work-up, including medical history, physical and neurological assessment, cognitive testing, MRI, laboratory tests, and in a subset examination of cerebrospinal fluid (CSF) (*n* = 883). Genetic testing for FTD genes where not performed as part of the standard assessment. Patients were diagnosed as SCD when the cognitive complaints were not accompanied by objectively confirmed cognitive impairment, and the criteria for MCI or dementia were not met (Albert et al. 2011; McKhann et al. 2011). The National Institute on Aging-Alzheimer's Association (NIA-AA) criteria were used to diagnose patients with MCI (Albert et al. 2011) and dementia due to AD (McKhann et al. 2011). The Neary and Snowden et al. or the Mckhann et al. criteria were used for FTD, and the Rascovsky et al. criteria for bvFTD and Gorno-Tempini et al. criteria for svPPA and nfvPPA (Gorno-Tempini et al. 2011; McKhann 2001; Neary et al. 1998; Rascovsky et al. 2011). VaD was diagnosed according to the NINDS-AIREN criteria (Román et al. 1993) and DLB to the McKeith criteria (Mckeith et al. 2005). Moreover, published criteria were used to diagnose Parkinson's disease with dementia (Emre et al. 2007), atypical parkinsonism (Armstrong et al. 2013; Gilman et al. 2008; Litvan et al. 1996), and normal pressure hydrocephalus (Relkin et al. 2005). All patients had as a minimum a 12-month clinical follow-up evaluation confirming the diagnosis. Moreover, all patients provided written informed consent for their data to be used for research purposes.

### 2.2 Image acquisition and quantification

MRI were acquired on 1 T, 1.5 T, or 3 T scanners (voxel size 0.5–1.0 × 0.5–1.0 × 0.5–1.5 mm). In all centres, availability was the key driver in assigning patients to different scanners. Seven patients were excluded from the study because of imaging artifacts or noise. The imaging biomarkers were extracted from T1-weighted images using a multi-atlas segmentation algorithm based on (Lötjönen et al. 2010). Using this method, the patient image and 79 atlases are first registered to a template image. The 28 atlases that are most similar to the patient image are identified and registered to the patient image using a dense non-rigid transformation. Finally, a probabilistic atlas is generated from the transformed atlases and used as a spatial prior model in the expectation maximization classification algorithm. Using this algorithm all the MRI images were segmented into 133 regions (102 cortical and 31 sub-cortical regions) of which volumes for 44 frontal, 24 temporal, 18 parietal, and 16 occipital lobe regions were used in the analysis. Of these 102 cortical regions half were from the left side and half from the right side.

Three MRI biomarkers were derived from the segmented volumes using z-scores. The first MRI biomarker, the anterior vs. posterior index (API), reflects the fact that frontal and temporal brain regions are affected in FTD, whereas posterior regions are relatively preserved. The index was defined as a z-score:

$$\mathrm{API}=\frac{\log V_{A}/V_{P}-\mu }{\sigma }$$

where V_A_ denotes the weighted volume of all brain regions in the frontal and temporal lobes and V_P_ in the parietal and occipital lobes. As the z-score should distinguish FTD from the other diagnostic groups, *μ* and *σ* are the average and standard deviation of log*V*_*A*_/*V*_*P*_ computed for all non-FTD patients.

The two most common early-onset causes of dementia, i.e. AD and FTD, were used to determine the weight for the volume of each region. The weight was defined as the difference in the average volume of the region between AD and FTD patients for the anterior regions and between FTD and AD patients for the posterior regions, divided by the average volume of the region for all non-FTD patients. If the weight was negative, it was set to zero. At visual inspection, the distribution of the regions with a high weight (>0.05) formed a compact region both in the anterior and posterior part of the brain (See Supplemental file, Fig. 1A). The only exceptions were the parahippocampal gyrus and the inferior temporal gyrus, and as outliers from the anatomic pattern these regions were excluded without affecting the performance of API significantly. Table 1A in the Supplemental file shows the weights for all cortical regions.

**Fig. 1:**
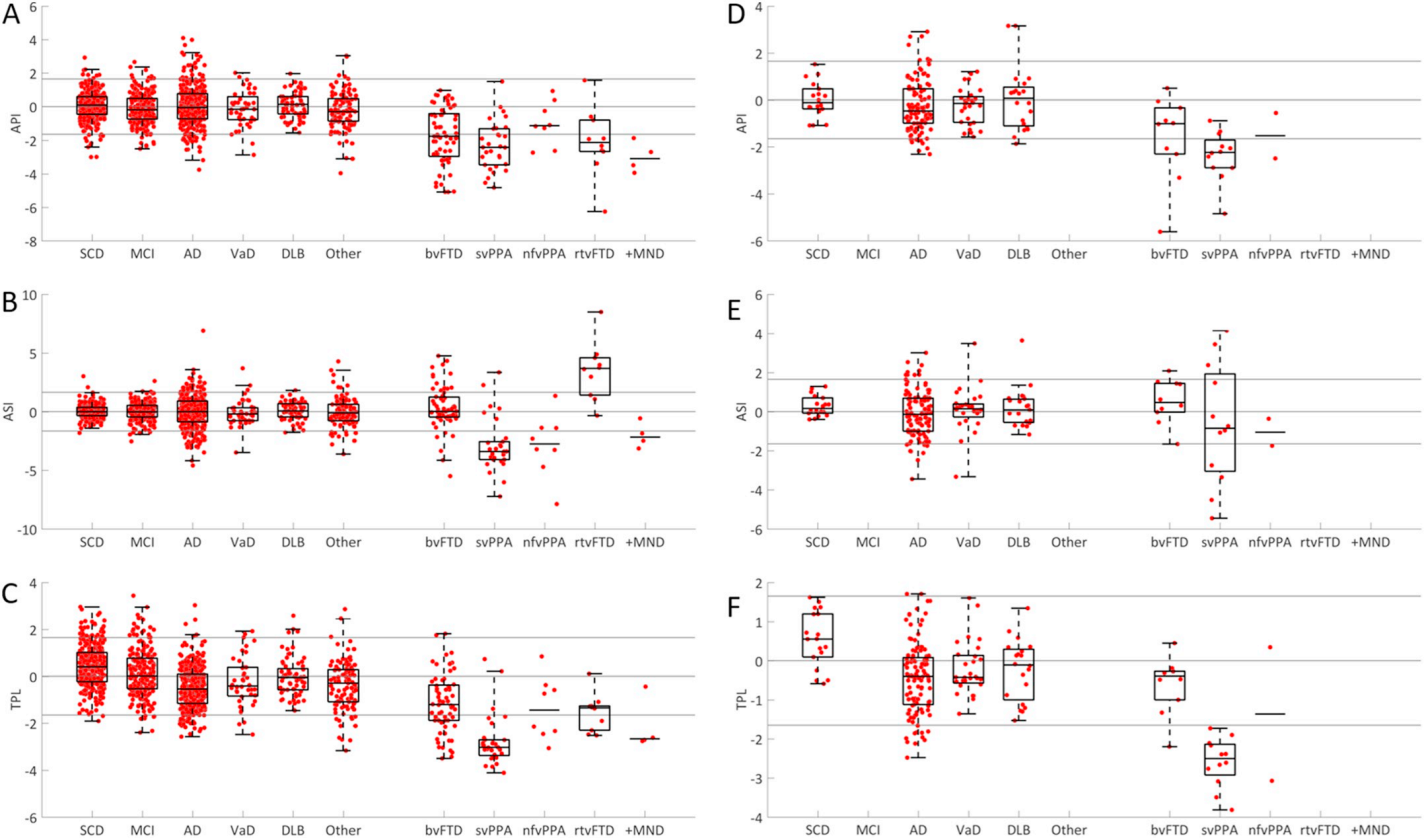
Scatter plots including box- and whiskers plots of API, ASI and TPL versus the clinical diagnoses. A–C) the ADC+ PredictND cohort and D-F) the DDRC cohort. The z-score of the imaging biomarker (y-axis) and clinical diagnosis (x-axis) for 1) the anterior vs. posterior index (API), 2) the asymmetric index (ASI), and 3) volume of the temporal pole left index (TPL). If the number of cases per groups is small, *n* ≤ 10, only median is shown. Abbreviations: SCD: subjective cognitive decline, MCI: mild cognitive impairment, AD: Alzheimer's disease, VaD: vascular dementia, DLB: dementia with Lewy bodies, Other: other dementias, bvFTD: behavior variant frontotemporal dementia, svPPA: semantic variant primary progressive aphasia, nfvPPA: non-fluent variant PPA, rtvFTD: right temporal variant FTD, +MND: FTD with motor neuron disease.

The second MRI biomarker, the asymmetry index (ASI), reflects the fact that atrophy is often asymmetric in FTD subtypes, such as svPPA and nfvPPA. The index was defined equally to API as a z-score:

$$\mathrm{ASI}=\frac{\log V_{L}/V_{R}-\mu }{\sigma }$$

where V_L_ denotes the weighted volume of all brain regions in the left frontal and temporal lobes and V_R_ in the right frontal and temporal lobes, and *μ* and *σ* are the average and standard deviation of log*V*_*L*_/*V*_*R*_ computed for all non-FTD. The weights were defined in the same way as for API.

Finally, the volume of each of the 133 cortical and sub-cortical regions was tested separately as an imaging biomarker. As the left temporal pole (TPL) in this analysis demonstrated highest discriminative performance, the third MRI biomarker was defined as the volume of the TPL (See Supplemental file, Table 2A). For consistency, TPL was transformed into z-scores in the same way as API and ASI.

### 2.3 Data analysis

We assessed differences in baseline characteristics between diagnostic groups using analysis of variance (ANOVA), Kruskal-Wallis tests, and Pearson χ2 tests when appropriate. All volumes were normalized first for the head size (Buckner et al. 2004), and then for age and sex (Cole and Green 1992). There were no missing MRI data or detection of outliers, and all data were used in all computations. Separate training and test sets using 10-fold cross-validation were used. Further, all three biomarkers were tested in the independent DDRC cohort.

First, we assessed the performance of the three imaging biomarkers for separation of FTD from all non-FTD diagnostic groups (AD, DLB, VaD, other dementias, MCI, and SCD), and from AD separately. Thereafter, we studied how well subtypes of FTD (bvFTD, svPPA, and svPPA+nfvPPA) were differentiated from one another. We repeated all analyses stratifying for age. A cut-off below/above 70 years was chosen to achieve maximum exclusion of other diagnostic groups while maintaining the highest number of FTD cases. We used the following performance metrics: sensitivity, specificity, and area under the receiver operating characteristic curve (AUC). To account for the low prevalence of FTD in clinical practice we chose a cut-off value that leads to a specificity about 95%. For normally distributed variables, such as API, a specificity of 95% is obtained with the cut-off value z = −1.65, i.e. all cases with API < −1.65 are classified as FTD in the analysis. When the classification of subtypes was studied, the constraint for high specificity was less relevant. In this analysis, an optimal cut-off value maximizing the average of sensitivity and specificity was defined.

Additionally, as 1 T images typically have lower signal-to-noise ration and contrast, we compared results with and without 1 T images. In addition to age and sex correction, we also studied the correction for the MRI field strength (1 T, 1.5 T, and 3 T). However, these additional analyses did not significant impact on the accuracy and are presented in the Supplemental file, Table 3-4A.

Finally, we explored differences between bvFTD cases with API < −1.65 versus API > −1.65 as an API value above the cut-off value indicates a less pronounced frontotemporal atrophy pattern. First, we explored the relationship between amyloid-β and API using scatter plots. Second, we used the cluster analysis approach described in (Whitwell et al. 2009) and (Ranasinghe et al. 2016) to investigate distinct anatomic patterns. The modulated gray matter (GM) volumes of 18 regions of interest (ROIs) were defined from the ADC + PredictND cohort (Ranasinghe et al. 2016). Thereafter, hierarchical agglomerative cluster analysis was used to generate the clusters of atrophy patterns for the bvFTD cases (Whitwell et al. 2009). We analyzed the GM atrophy patterns of the bvFTD clusters relative to SCD and AD. All the analyses were performed using MATLAB version R2015b (MathWorks, Natick, MA, USA).

## 3 Results

### 3.1 Patients

The baseline characteristics of the ADC + PredictND and DDRC cohorts, including FTD subtypes, are presented in Table 1 (See Supplemental file for additional details, Table 5A).

**Table 1:** Baseline characteristics of the ADC + PredictND and DDRC cohort.

		non-FTD					
	FTD	AD	DLB	VaD	Other	MCI	SCD
A) All cases							
**ADC + PredictND** (*n* = 1213)							
Number of patients	116	341	66	40	104	229	317
Female, n (%)	50 (43)	190 (56)††	10 (15)†	15 (38)	53 (51)	87 (38)	173 (55)††
Age (years), mean (SD)	64 (7)†	68 (8)	69 (8)	71 (8)	73 (9)ⱡ	67 (8)	62 (9)†
MMSE, mean (SD)	24 (5)	22 (5)†	24 (4)	24 (4)	25 (4)	27 (2)†	29 (1)†
AB42, pg/ml	881 (291)†	531 (167)†	730 (258)	700 (263)	729 (335)	751 (310)	920 (251)†
Total tau, pg/ml	395 (260)	695 (407)†	341 (213)	305 (160)	480 (271)	443 (260)	290 (166)††
P tau, pg/ml	51 (25)	86 (39)†	51 (27)	44 (20)	65 (31)	66 (33)	49 (20)††
**DDRC** (n = 200)							
Number of patients	24	110	20	28	0	0	18
Female, n (%)	9 (38)	68 (62)†	7 (35)	11 (39)	–	–	6 (33)
Age (years), mean (SD)	68 (10)	72 (10)	72 (9)	72 (8)	–	–	63 (7)†
MMSE, mean (SD)	25 (4)	24 (4)	24 (4)	25 (4)	–	–	29(2)†


	FTD				
	bvFTD	svPPA	rtvFTD	nfvPPA	+MND
B) FTD cases					
**ADC + PredictND** (*n* = 116)					
Number of patients	64	30	10	8	4
Female, n. (%)	28 (44)	14 (47)	3 (30)	3 (38)	2 (50)
Age (years), mean (SD)	63 (7)	63 (6)	63 (7)	70 (6)	62 (6)
MMSE, mean (SD)	24 (4)	22 (6)	27 (3)	23 (7)	22 (5)
AB42, pg/ml	923 (269)⁎	780 (281)	955 (297)	685 (385)	1123 (249)
Total tau, pg/ml	390 (307)	387 (216)	385 (106)	452 (236)	470 (177)
P tau, pg/ml	51 (28)	52 (26)	45 (13)	62 (23)	39 (6)
**DDRC** (*n* = 24)					
Number of patients	10	12	0	2	0
Female, n. (%)	3 (30)	5 (42)	–	1 (50)	–
Age (years), mean (SD)	65 (13)	69 (7)	–	72 (8)	–
MMSE, mean (SD)	25 (5)	25 (5)	–	23 (1)	–

Abbreviations: FTD: frontotemporal dementia, AD: Alzheimer's disease, DLB: dementia with Lewy bodies, VaD: vascular dementia, Other: other dementias, MCI: mild cognitive impairment, SCD: subjective cognitive decline, MMSE: the mini mental state examination, bvFTD: behavioral variant FTD, svPPA: semantic variant primary progressive aphasia, rvtFTD: right temporal variant FTD, nfvPPA: non-fluent variant PPA, +MND: FTD + motor neuron disease.

† Differ significantly from all other groups (p < 0.05).

†† Differ significantly from MCI and VaD (p < 0.05).

ⱡ Differ from MCI, AD and DLB.

⁎ Differ from svPPA.

### 3.2 Performance of API, ASI, and TPL

Table 2 shows sensitivity, specificity, and AUC computed for the ADC + PredictND cohort for all patients and for patients below 70 years of age. Only the imaging biomarkers with the highest performance for each classification are presented (See Supplemental file for additional results, Tables 2A, 6-7A). At the required specificity close to 95%, API separated FTD from non-FTD with a sensitivity of 59% (AUC = 83%) (See Supplemental file for performances at other specificities, Table 8A). When separating FTD from AD the performance of API was almost the same (AUC = 82%). Further, we found that API performed with slightly higher sensitivity (63%) and AUC (87% for non-FTD and 88% for AD) when applied to patients below 70 years. ASI performed with 79% sensitivity and 92% specificity (AUC = 85%) for separation of svPPA+nfvPPA from bvFTD, whereas TPL separated svPPA from bvFTD with 82% sensitivity and 80% specificity (AUC = 85%). For the same diagnostic comparisons ASI performed slightly better (AUC = 88%) and TPL slightly worse (AUC = 83%) in the cohort <70 years.

**Table 2:** Sensitivity, specificity and AUC computed for the ADC + PredictND and DDRC Cohort.

ADC + PredictND	ALL (n = 1213)			<70 years (*n* = 771)			Imaging biomarker
	sens	spec	AUC	sens	spec	AUC	
FTD vs. non-FTD	0.59	0.95	0.83	0.63	0.96	0.87	API
FTD vs. AD	0.59	0.93	0.82	0.63	0.95	0.88	API
svPPA+nfvPPA vs. bvFTD	0.79	0.92	0.85	0.85	0.91	0.88	ASI
svPPA+nfvPPA vs. bvFTD	0.74	0.80	0.78	0.76	0.77	0.79	TPL
svPPA vs. bvFTD	0.80	0.93	0.85	0.84	0.91	0.87	ASI
svPPA vs. bvFTD	0.82	0.80	0.85	0.82	0.77	0.83	TPL
**DDRC**	ALL (n = 200)			<70 years (*n* = 72)			

sens	spec	AUC	sens	spec	AUC

FTD vs. non-FTD	0.58	0.94	0.85	0.64	0.93	0.93	API
FTD vs. AD	0.58	0.92	0.84	0.64	0.89	0.93	API
svPPA+nfvPPA vs. bvFTD	0.29	1.00	0.68	0.43	1.00	0.86	ASI
svPPA+nfvPPA vs. bvFTD	0.79	0.90	0.91	1.00	1.00	1.00	TPL
svPPA vs. bvFTD	0.33	1.00	0.64	0.50	1.00	0.83	ASI
svPPA vs. bvFTD	0.83	0.90	0.97	1.00	1.00	1.00	TPL

Abbreviations: Sens: sensitivity, spec: specificity, AUC: the area under the receiver operating characteristic curve, FTD: frontotemporal dementia, Non-FTD: all other diagnostic groups, bvFTD: behavioral variant FTD, svPPA: semantic variant primary progressive aphasia, nfvPPA: non-fluent variant primary progressive aphasia, API: the anterior vs. posterior index, ASI: the asymmetric index, TPL: the temporal lobe left index.

The table presents the sensitivity, specificity and AUC in the ADC + PredictND and DDRC cohort. The results are shown for all patients, and patients <70 years. Only the imaging biomarkers with the highest performance for the relevant comparisons are presented (last column).

When the model was applied to the independent DDRC cohort, the performance of API for detection of FTD from non-FTD (AUC = 85%) and TPL for separation of svPPA from bvFTD (AUC = 97%) were comparable to the original results (Table 2). In contrast, the performance of ASI for separation of svPPA+nfvPPA from bvFTD decreased from an AUC of 85% to 68% (Table 2).

Scatter plots including box-and-whiskers plots for the different imaging biomarkers and diagnostics groups are shown in Fig. 1. The plots show that API provides high separation of FTD from non-FTD but little separation with regards to FTD subtypes. Both ASI and TPL perform better than API with regards to separation of the FTD subtypes svPPA and nfvPPA.

**Fig. 2:**
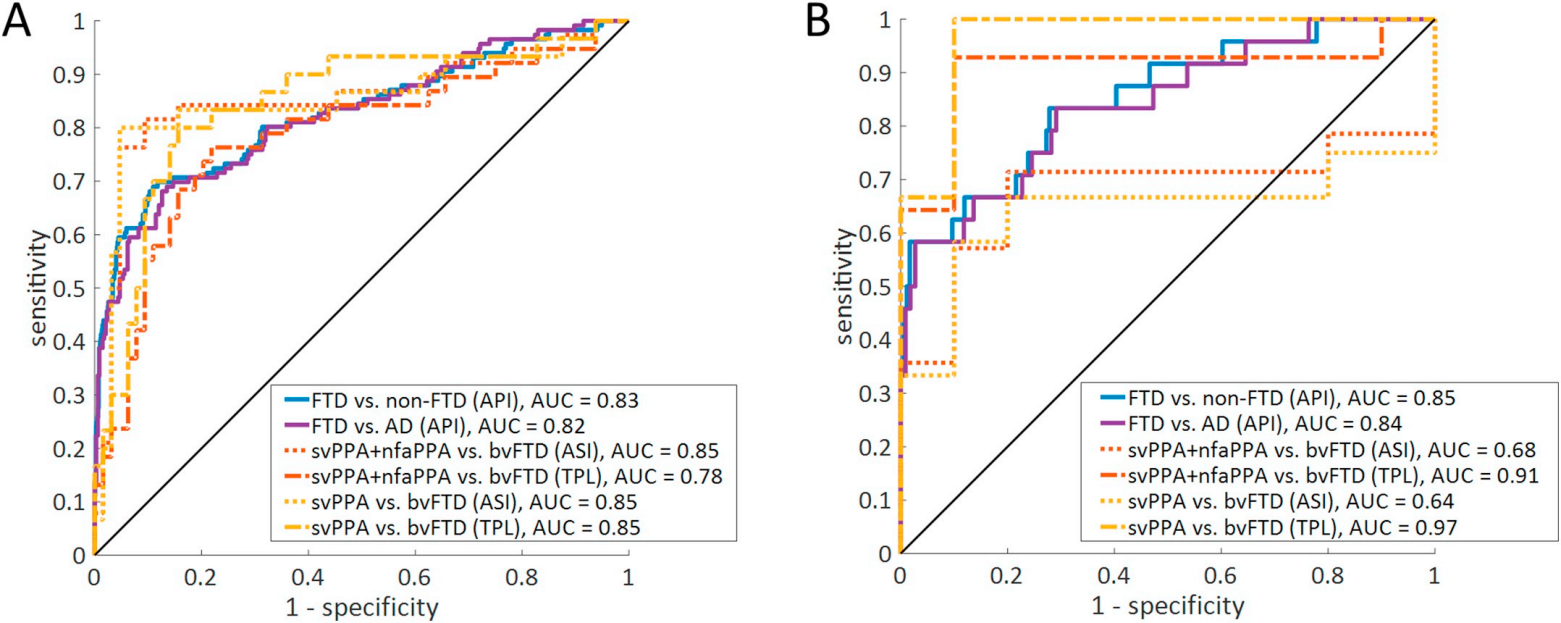
Area under the ROC for API, ASI and TPL. A) the ADC + PredictND cohort and A) the DDRC cohort. AUC values are displayed for each comparison. Abbreviations: ROC: the receiver operator characteristic, FTD: Frontotemporal dementia, svPPA: semantic variant primary progressive aphasia, nfvPPA: non-fluent variant PPA.

Fig. 2 shows the receiver operating characteristic (ROC) curves for the ADC + PredictND cohort and DDRC cohort.

### 3.3 Exploration of bvFTD cases with API values above the cut-off value

**Fig. 3:**
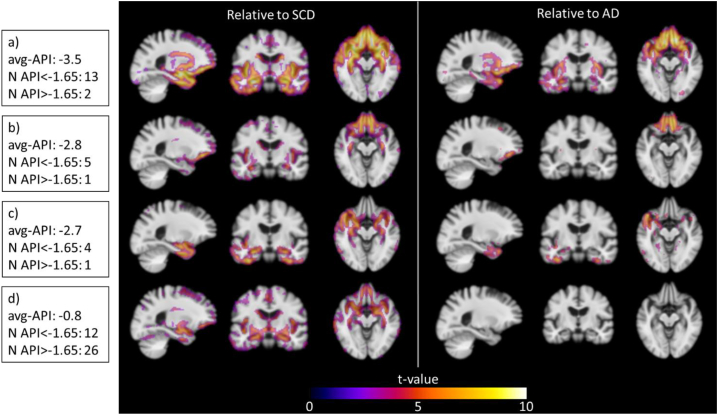
Cluster analysis of anatomic patterns in the bvFTD group, the ADC + PredictND cohort. Gray matter atrophy patterns of the bvFTD clusters relative to patients with SCD and AD patients (p < 0.05, false discovery rate correction for multiple comparisons) based on the modulated gray matter volumes from voxel-based morphometry. The analysis defines a network of degeneration for 4 groups: a) frontal and temporal gray matter loss with subcortical involvement, b) frontal atrophy and subcortical involvement, c) temporal and modest subcortical involvement and d) minimal cortical and predominantly subcortical atrophy. The number of patients with API < −1.65 and API > −1.65 is presented for each group. Abbreviations: AD: Alzheimer's disease, SCD: subjective cognitive decline, avg-API: average Anterior vs. Posterior Index.

An API value above the cut-off value indicating less pronounced frontotemporal atrophy pattern was observed in half of the bvFTD cases. Data showed a difference in age, trail making test B (TMT) and total cortical gray matter volume (CGM) between the group of bvFTD cases below the cut-off value compared to the group above: API < −1.65 (*n* = 32, age: 62 ± 6, TMT-B: 169 ± 184 s, CGM: 429 ± 36 ml) and API > −1.65 (n = 32, age: 65 ± 8 (*p* = 0.04), TMT-B: 238 ± 125 s (*p* = 0.004), CGM: 480 ± 42 ml (*p* = 0.02)) (See Supplemental file, Table 9A). When exploring the relationship between amyloid-β and API, we found no difference between the two groups in terms of CSF findings reflecting amyloid pathology (See Supplemental file, Fig. 2A). Thereafter, a cluster analysis was performed to assess heterogeneity of anatomic patterns in the bvFTD cases as shown in Fig. 3. When comparing bvFTD with SCD we identified 4 clusters: a) frontal and temporal gray matter loss with subcortical involvement, b) frontal atrophy and subcortical involvement, c) temporal and modest subcortical involvement, and d) predominantly subcortical atrophy. Moreover, the result showed that the subcortical cluster group contained >80% of the API > −1.65 cases. When comparing bvFTD and AD we found no differences between the subcortical bvFTD and AD cases demonstrating a very similar atrophy pattern for these two groups.

## 4 Discussion

In this retrospective multicenter study, we developed three diagnostic imaging biomarkers (API, ASI, and TPL), derived from volumetric MRI, for diagnosis of FTD and separation of FTD subtypes. API performed with an AUC of 83% and 82% when detecting FTD from non-FTD and AD patients, respectively. A distinct frontotemporal atrophy pattern was detectable in 59% of all FTD patients, whereas a large proportion of the remaining FTD cases demonstrated a subcortical atrophy pattern similar to AD. ASI showed highest performance for detecting the two PPA subtypes, whereas TPL was specific for detection of svPPA.

Since the prevalence of FTD is relatively low (accounts for approximately 3% of all dementia and 10% of early-onset dementia), an imaging biomarker should perform with a high specificity to avoid false positive FTD diagnoses in clinical practice (Hogan et al. 2016). For detection of FTD using API we therefore aimed for a high specificity with the trade-off of a moderate-low sensitivity, although still comparable to other MRI derived biomarkers with lower specificity (Bouts et al. 2018; Harper et al. 2016; Muñoz-Ruiz et al. 2012; Steketee et al. 2016a). Compared to API, studies with advanced MRI methods, such as voxel-based morphometry, resting state functional MRI, diffusion tensor imaging (DTI), and arterial spin labelling (ASL) have for single imaging biomarkers shown equal or lower AUC ranging from 61 to 81% (Accuracy: 72–79%, Sensitivity: 60–83%, Specificity: 63–93%) when differentiating between AD and FTD (Bouts et al. 2018; Bron et al. 2017; Cajanus et al. 2018; Canu et al. 2017; Klöppel et al. 2015; Möller et al. 2015b; Muñoz-Ruiz et al. 2012; Steketee et al. 2016a). Our study presents relatively simple MRI atrophy biomarkers readily applicable to clinical practice as an additional tool in the clinicians´ armamentarium. Supporting this, in another study comparing the performance of different commonly used diagnostic tests (cognitive tests, CSF biomarkers, and automated MRI features) we found that API performed at comparable or better levels than most tests for separation of FTD from other dementia groups (Bruun et al. 2018).

Separating bvFTD from AD is often difficult (Mendez et al. 2013; Neary et al. 1998). In the present study, the performance of API when separating FTD from non-FTD and from AD was comparable. However, visually the scatter plots show a smaller difference between bvFTD and AD. This may be due to the fact that both groups may present with atrophy in the temporal lobe and that atypical atrophy patterns are frequent in AD (Lehmann et al. 2012; Ossenkoppele et al. 2015; van de Pol 2006). Furthermore, a frontal variant of AD also exist (McKhann et al. 2011). The performance of API increased when applied only to patients below 70 years of age indicating that the index might perform better in this subgroup. This could be due to the fact that AD more often presents as posterior cortical atrophy in the younger AD population, or that focal atrophy may be attenuated by the effect of age-related global atrophy in older adults (Ge et al. 2002; Steketee et al. 2016a).

Only a few studies using imaging biomarkers have addressed subtyping of FTD (Agosta et al. 2015; Canu et al. 2017; Meyer et al. 2017; Möller et al. 2015b, 2015a), although correct classification of subtypes is important regarding prognosis, supportive measures, and patient information. In this study, we found high performance of ASI and TPL for separation of svPPA+nfvPPA from bvFTD, and specifically TPL for detection of svPPA. However, due to the small numbers of nfvPPA the results combining svPPA+nfvPPA are subject to some uncertainty and must be interpreted with caution. Nevertheless, the atrophy patterns of FTD subtypes with predominantly asymmetrical affection in nfvPPA and svPPA, and temporal affection in svPPA seem to be a rational approach for separation of PPA subtypes from non-FTD and bvFTD (Gorno-Tempini et al. 2004; Schroeter et al. 2007).

When validating the three biomarkers in the independent DDRC cohort the result for API and TPL seemed to be generalizable. In contrast, for ASI the performance decreased. However, this could as well be due to the small number of FTD cases in the DDRC cohort rather than lack of generalization and should be retested in a larger FTD cohort.

A considerable number of bvFTD cases presented without severe frontotemporal atrophy (API above the cut-off value, i.e. false negative cases). The diagnostic uncertainty when using clinical diagnoses without post-mortem confirmation of the underlying neuropathology and co-occurrence of other pathologies, should be considered (Brunnström and Englund 2009; Mendez et al. 2013; Toledo et al. 2012). No difference in the relationship between amyloid-β and API was though found between the bvFTD group with API values below compared to above the cut-off value suggesting that misdiagnosis and dual pathology are not the main reason for the false negative cases. Contamination with FTD phenocopies should also be considered (Steketee et al. 2016b). However, the most likely explanation is heterogeneity, i.e. that depending on pathological and genetic subtypes the atrophy patterns vary (Cash et al. 2018; Rohrer et al. 2011; Whitwell et al. 2012, 2009). Moreover, previous studies have found that a considerable proportion of bvFTD does not have atrophy detectable on MRI (Kipps et al. 2009, 2007). We performed a cluster analysis and based on atrophy patterns identified 4 subtypes similar to what has previously been described (Ranasinghe et al. 2016), showing that the bvFTD cases with API > −1.65 were predominantly of the subcortical atrophy subtype. Since API is derived from cortical atrophy patterns, it is not ideal for detection of subcortical atrophy. Interestingly, our results showed that the subcortical bvFTD subgroup had an atrophy pattern similar to AD, which should be taken into account in the development of MRI-based imaging biomarkers for these FTD cases. Future clinical imaging biomarker studies should consider other approaches, e.g., repeated scans or other data, to capture this bvFTD subgroup with a more AD-like subcortical atrophy pattern.

A strength of our study is the large ADC + PredictND multicenter cohort containing a spectrum of diagnostic groups which is relatively representative of the most frequent diagnosis in a memory clinic. However, a potentially slightly higher prevalence of the less common diseases, e.g., FTD and DLB, might have influence the result and additional validation in a prospectively recruited cohort should be pursued. The multicenter data was collected from >15 different scanners which precluded us from making comparisons across scanners. However, the use of different scanners might add to the generalizability of the biomarkers. Another limitation of the study was the relatively few nfvPPA and svPPA cases making it difficult to validate the TPL and ASI biomarkers for each subtype individually. Moreover, the independent validation cohort was relatively small, and to maximize the number of FTD cases for the age stratification a cut-off of 70 years was chosen rather than the more commonly used definition of early-onset dementia (i.e., 65 years). These issues underline the importance of further validation in a larger FTD cohort with especially more PPA cases. Another issue for consideration is the possibility of circularity. In this study, MRI scans had been used in the determination of the clinical diagnosis. However, the derived automated MRI biomarkers were computed for the study and had not been used for clinical diagnosis. Finally, the clinical diagnosis is associated with some restraints due to a known degree of discrepancy between clinical and post-mortem neuropathological diagnoses (Brunnström and Englund 2009; Mendez et al. 2013; Schneider et al. 2009). Further, information regarding the main FTD genes were not available for the cohorts. Performance of the biomarkers using the neuropathological diagnosis or genetic status would therefore be interesting to explore in future research (Cash et al. 2018; Rohrer et al. 2011; Whitwell et al. 2012).

In conclusion, the presented MRI biomarkers were developed on the prerequisite of clinically relevant high specificity, and API was able to detect a distinct frontotemporal MRI pattern in 59% of all FTD patients. Moreover, our results suggest that a considerable number of the undetectable bvFTD cases had a non-specific subcortical MRI pattern similar to AD, and other approaches than MRI might be needed to detect this subgroup. Finally, we found that ASI could aid in detecting svPPA and nfvPPA, whereas TPL was more specific for svPPA. The three biomarkers are applicable for clinical use and could provide additional information to the diagnostic assessment and improve differential diagnosis of FTD in clinical practice.

## Appendix A Supplementary data

Supplementary material Image 1
