# Supplementary material for: Detecting frontotemporal dementia syndromes using MRI biomarkers
Source: Neuroimage Clin. 2019 Feb 4;22:101711. doi: 10.1016/j.nicl.2019.101711 (PMC6369219; doi:10.1016/j.nicl.2019.101711)
Supplement: Supplementary file 1 — Supplementary material [file mmc1.docx]

**SUPPLEMENTAL FILE**

**Figure 1A. Illustration of the brain regions with a high weight (>0.05) when computing the anterior vs. posterior index (API).**

| 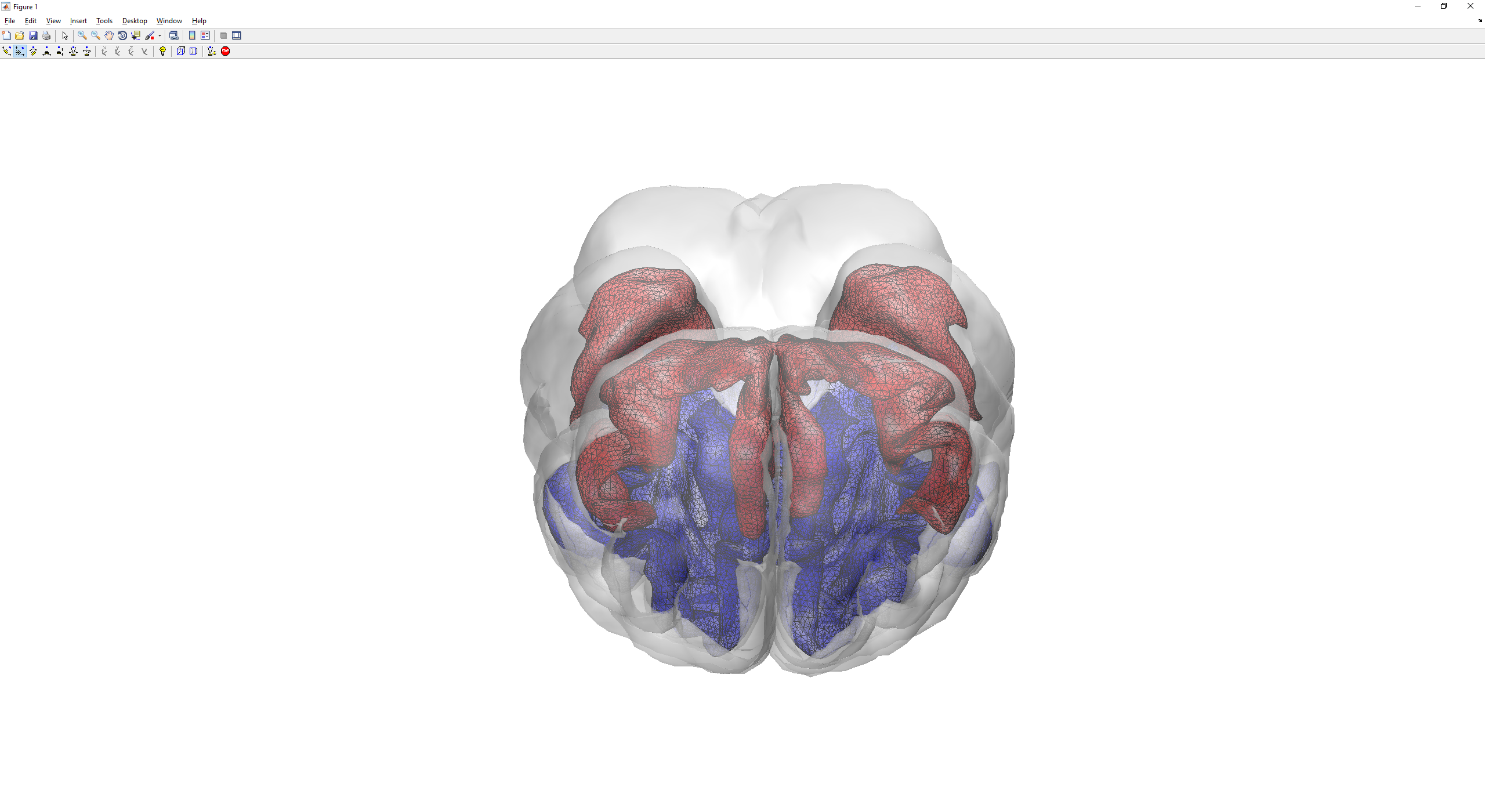 | 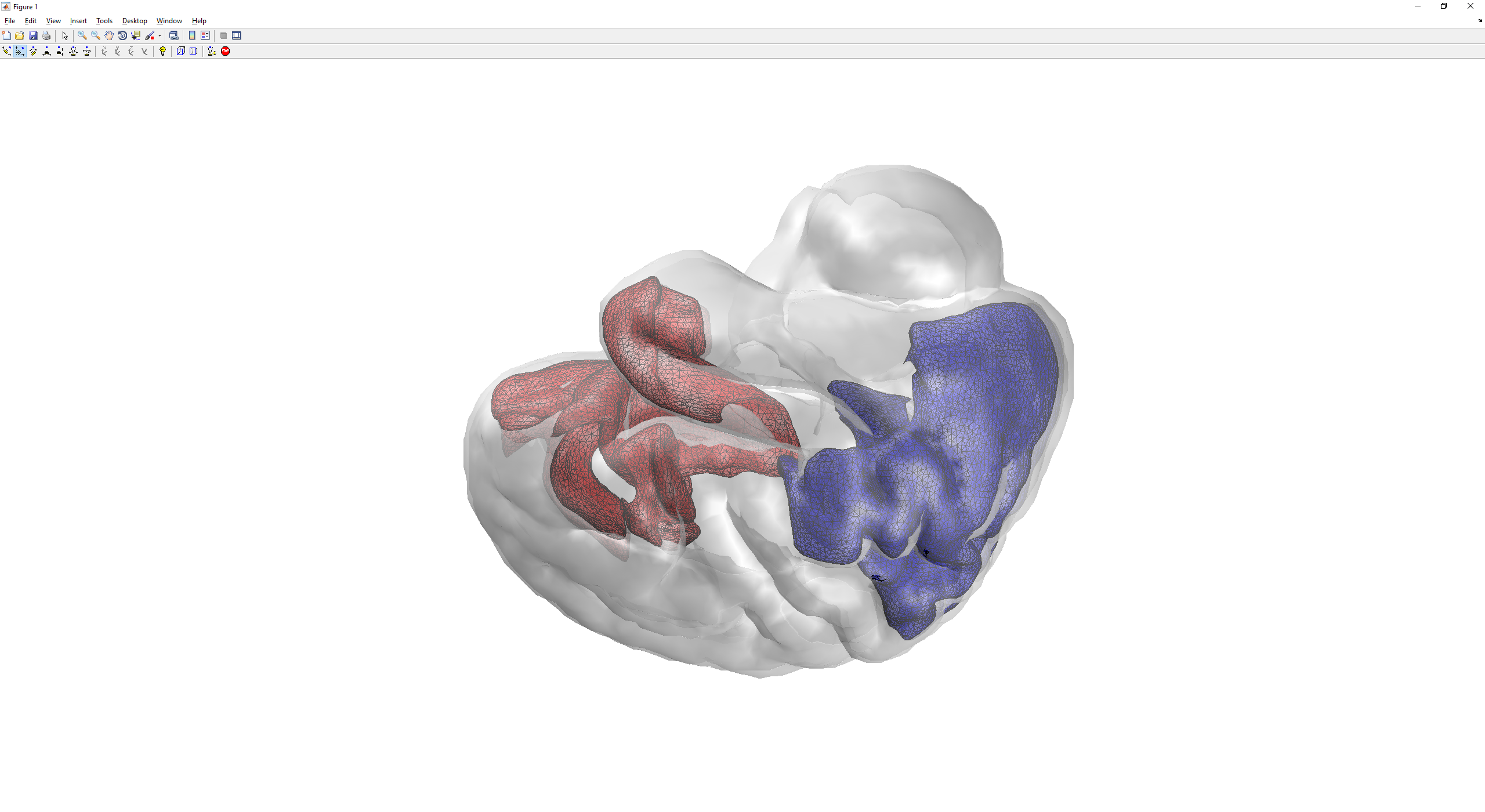 | 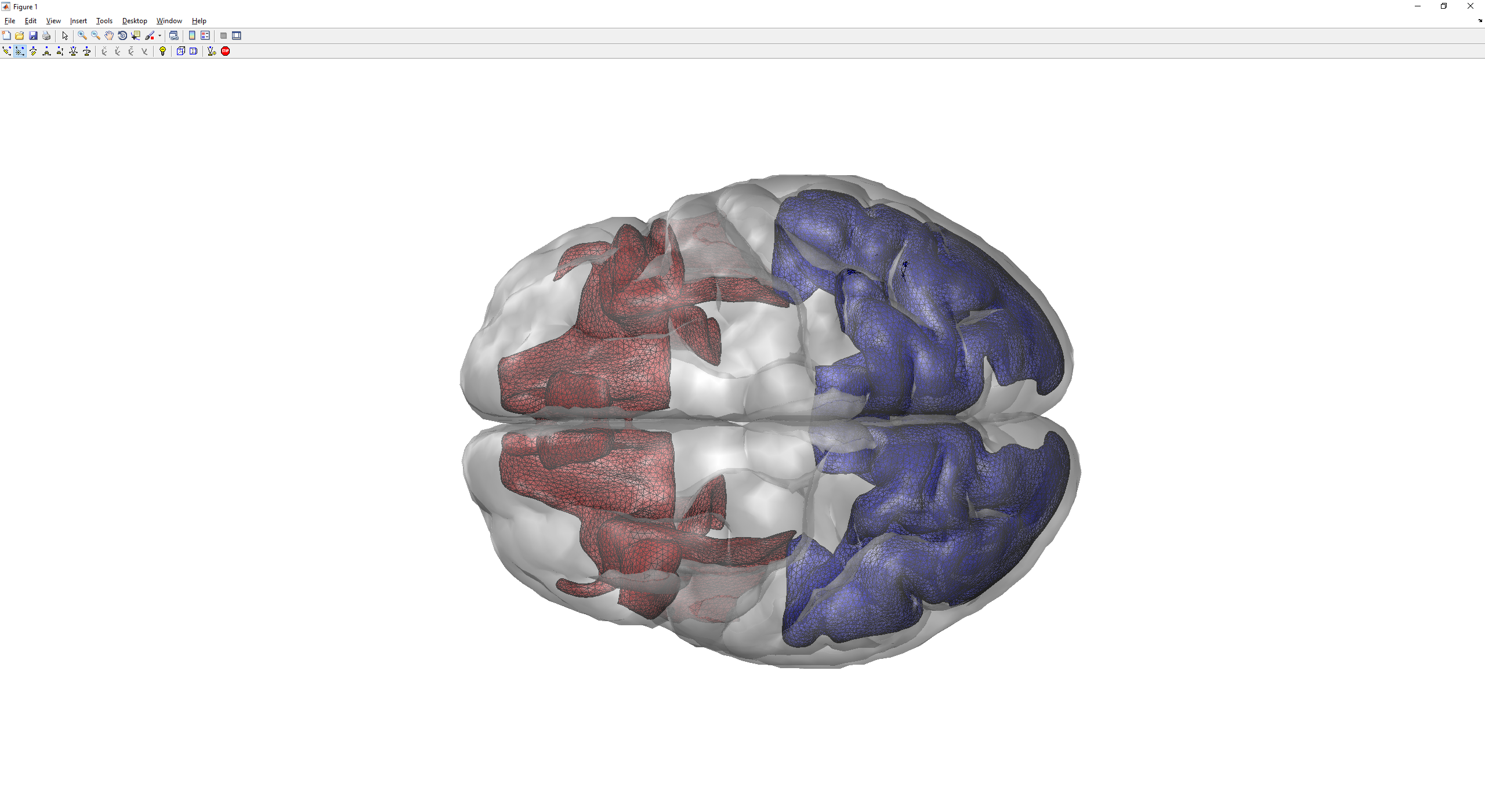 |
| --- | --- | --- |

The red color denotes regions in the frontal and temporal lobes where difference of the average volume between FTD and AD patients is higher than 5% of the average volume of the region. Likewise, the blue color denotes regions with a weight higher than 0.05 in parietal and occipital lobes.

**Table 1A. The weight of all cortical regions**

| Lobe | Region | w(API) | w(ASI) |  | Lobe | Region | w(API) | w(ASI) |
| --- | --- | --- | --- | --- | --- | --- | --- | --- |
| FL | Anterior Cingulate Gyrus | 0.087 | 0.110 |  | PL | Angular Gyrus | 0.020 | 0.049 |
| FL | Anterior Insula | 0.143 | 0.177 |  | PL | Parietal Operculum | 0.022 | 0.094 |
| FL | Anterior Orbital Gyrus | 0.043 | 0.103 |  | PL | Posterior Cingulate Gyrus | 0.052 | 0.066 |
| FL | Basal Forebrain | 0.112 | 0 |  | PL | Postcentral Gyrus | 0.016 | 0 |
| FL | Central Operculum | 0.027 | 0.015 |  | PL | Postcentral Gyrus Med. Segm. | 0 | 0 |
| FL | Frontal Operculum | 0.097 | 0.031 |  | PL | Precuneus | 0.044 | 0.027 |
| FL | Frontal Pole | 0 | 0.010 |  | PL | Superior Parietal Lobule | 0.035 | 0.024 |
| FL | Gyrus Rectus | 0.106 | 0.089 |  | PL | Supramarginal Gyrus | 0.054 | 0.085 |
| FL | Lateral Orbital Gyrus | 0.061 | 0.141 |  | OL | Calcarine Cortex | 0 | 0.019 |
| FL | Medial Frontal Cortex | 0.097 | 0.138 |  | OL | Cuneus | 0 | 0.073 |
| FL | Medial Orbital Gyrus | 0.087 | 0.096 |  | OL | Inf. Occipital Gyrus | 0.028 | 0.033 |
| FL | Middle Cingulate Gyrus | 0.008 | 0.074 |  | OL | Lingual Gyrus | 0 | 0.048 |
| FL | Middle Frontal Gyrus | 0.024 | 0.035 |  | OL | Middle Occipital Gyrus | 0.025 | 0.053 |
| FL | Operc. Part Inf. Frontal Gyrus | 0.092 | 0.090 |  | OL | Occipital Fusiform Gyrus | 0.006 | 0.042 |
| FL | Orbital Part Inf. Frontal Gyrus | 0.090 | 0.074 |  | OL | Occipital Pole | 0.070 | 0 |
| FL | Posterior Insula | 0.080 | 0.121 |  | OL | Superior Occipital Gyrus | 0.039 | 0 |
| FL | Posterior Orbital Gyrus | 0.099 | 0.155 |  |  |  |  |  |
| FL | Precentral Gyrus | 0.031 | 0.013 |  |  |  |  |  |
| FL | Precentral Gyrus Medial Segm. | 0.030 | 0.024 |  |  |  |  |  |
| FL | Subcallosal Area | 0.116 | 0.105 |  |  |  |  |  |
| FL | Sup. Frontal Gyrus | 0.027 | 0.055 |  |  |  |  |  |
| FL | Sup. Frontal Gyrus Med. Segm. | 0.075 | 0 |  |  |  |  |  |
| FL | Supplementary Motor Cortex | 0.062 | 0.030 |  |  |  |  |  |
| FL | Triang. Part Inf. Frontal Gyrus | 0.073 | 0.024 |  |  |  |  |  |
| TL | Amygdala | 0.117 | 0.243 |  |  |  |  |  |
| TL | Entorhinal Area | 0.129 | 0.211 |  |  |  |  |  |
| TL | Fusiform Gyrus | 0.081 | 0.197 |  |  |  |  |  |
| TL | Hippocampus | 0.044 | 0.190 |  |  |  |  |  |
| TL | Inferior Temporal Gyrus | 0.101 | 0.269 |  |  |  |  |  |
| TL | Middle Temporal Gyrus | 0.039 | 0.229 |  |  |  |  |  |
| TL | Parahippocampal Gyrus | 0.088 | 0.216 |  |  |  |  |  |
| TL | Planum Polare | 0.090 | 0.181 |  |  |  |  |  |
| TL | Planum Temporale | 0 | 0 |  |  |  |  |  |
| TL | Superior Temporal Gyrus | 0.015 | 0.143 |  |  |  |  |  |
| TL | Temporal Pole | 0.196 | 0.289 |  |  |  |  |  |
| TL | Transverse Temporal Gyrus | 0 | 0.129 |  |  |  |  |  |

The weight for the volume of each region was defined by comparing differences in the FTD and AD groups (API: anterior vs posterior regions, ASI: left vs right frontal and temporal lobes) divided by the average volume of the region for all subjects from all diagnostic groups.

Abbreviations: FL: Frontal lobe, TL: Temporal lobe, PL: Parietal lobe, OL: occipital lobe, API: the anterior vs. posterior index, ASI: the asymmetric index.

**Table 2A. AUCs for the FTD vs. non-FTD comparison for all regions.**

| Region | AUC |
| --- | --- |
| Temporal pole; left | 0.822 |
| Temporal pole; right | 0.800 |
| Anterior insula; left | 0.797 |
| Temporal lobe; left | 0.776 |
| Medial temporal lobe; left | 0.774 |
| Entorhinal area; left | 0.772 |
| Amygdala; left | 0.770 |
| Accumbens area; left | 0.769 |
| Anterior insula; right | 0.756 |
| Parahippocampal gyrus; left | 0.754 |
| Cerebral gray matter; left | 0.744 |
| Temporal lobe; right | 0.739 |
| Cerebral gray matter; total | 0.739 |
| Cerebral cortex; left | 0.738 |
| Entorhinal area; right | 0.738 |
| Accumbens area; right | 0.738 |
| Cerebral cortex; total | 0.733 |
| Posterior insula; left | 0.732 |
| Hippocampus; left | 0.728 |
| Amygdala; right | 0.727 |
| Inferior lateral ventricle; left | 0.725 |
| Caudate; left | 0.719 |
| Medial temporal lobe; right | 0.717 |
| Inferior temporal gyrus; right | 0.712 |
| Frontal lobe; left | 0.712 |
| Anterior cingulate gyrus; left | 0.712 |
| Planum polare; left | 0.711 |
| Frontal lobe; right | 0.706 |
| Fusiform gyrus; left | 0.705 |
| Gyrus rectus; left | 0.704 |
| Inferior temporal gyrus; left | 0.703 |
| Caudate; right | 0.703 |
| Posterior orbital gyrus; left | 0.701 |
| Basal forebrain; left | 0.699 |
| Medial frontal cortex; left | 0.697 |
| Superior frontal gyrus medial segment; right | 0.697 |
| Cerebral gray matter; right | 0.696 |
| Putamen; left | 0.693 |
| Cerebral cortex; right | 0.692 |
| Gyrus rectus; right | 0.692 |
| Medial orbital gyrus; left | 0.690 |
| Middle temporal gyrus; left | 0.689 |
| Parahippocampal gyrus; right | 0.686 |
| Ventral diencephalon; left | 0.682 |
| Posterior insula; right | 0.682 |
| 3rd ventricle; total | 0.679 |
| Fusiform gyrus; right | 0.675 |
| Basal forebrain; right | 0.674 |
| Frontal operculum; right | 0.673 |
| Medial frontal cortex; right | 0.672 |
| Medial orbital gyrus; right | 0.670 |
| Putamen; right | 0.670 |
| Hippocampus; right | 0.667 |
| Lateral orbital gyrus; left | 0.660 |
| Inferior lateral ventricle; right | 0.657 |
| Middle temporal gyrus; right | 0.657 |
| Posterior orbital gyrus; right | 0.654 |
| Subcallosal area; left | 0.654 |
| Supplementary motor cortex; right | 0.651 |
| Supplementary motor cortex; left | 0.647 |
| Cerebellar vermal lobules VIII-X; total | 0.646 |
| Frontal operculum; left | 0.646 |
| Cerebellar vermal lobules VI-VII; total | 0.644 |
| Superior frontal gyrus medial segment; left | 0.643 |
| Ventral diencephalon; right | 0.643 |
| Opercular part of the inferior frontal gyrus; right | 0.642 |
| CSFnm; total | 0.640 |
| Planum polare; right | 0.639 |
| Triangular part of the inferior frontal gyrus; right | 0.639 |
| Anterior cingulate gyrus; right | 0.639 |
| Thalamus proper; left | 0.637 |
| Cerebrospinal fluid; total | 0.636 |
| Superior temporal gyrus; left | 0.636 |
| Opercular part of the inferior frontal gyrus; left | 0.634 |
| Cerebellar vermal lobules I-V; total | 0.623 |
| Superior frontal gyrus; left | 0.621 |
| Lateral ventricle; left | 0.620 |
| Subcallosal area; right | 0.618 |
| Middle frontal gyrus; left | 0.614 |
| Orbital part of the inferior frontal gyrus; right | 0.612 |
| Middle cingulate gyrus; left | 0.610 |
| Middle frontal gyrus; right | 0.607 |
| Pallidum; right | 0.607 |
| Lateral orbital gyrus; right | 0.606 |
| Anterior orbital gyrus; left | 0.604 |
| Thalamus proper; right | 0.604 |
| Central operculum; right | 0.601 |
| Anterior orbital gyrus; right | 0.599 |
| Orbital part of the inferior frontal gyrus; left | 0.598 |
| Triangular part of the inferior frontal gyrus; left | 0.598 |
| Lateral ventricle; right | 0.593 |
| Central operculum; left | 0.591 |
| Precentral gyrus; left | 0.590 |
| Pallidum; left | 0.589 |
| Precentral gyrus medial segment; right | 0.589 |
| Superior occipital gyrus; left | 0.588 |
| Superior frontal gyrus; right | 0.586 |
| Middle occipital gyrus; left | 0.584 |
| Superior temporal gyrus; right | 0.582 |
| Lingual gyrus; left | 0.580 |
| Precentral gyrus; right | 0.573 |
| Cerebral white matter; left | 0.573 |
| Superior occipital gyrus; right | 0.573 |
| Inferior occipital gyrus; right | 0.571 |
| Cerebral white matter; total | 0.570 |
| Angular gyrus; right | 0.569 |
| Angular gyrus; left | 0.569 |
| Vessel; left | 0.569 |
| Occipital fusiform gyrus; right | 0.567 |
| Occipital lobe; left | 0.565 |
| Cerebral white matter; right | 0.565 |
| Occipital fusiform gyrus; left | 0.564 |
| Brain stem; total | 0.558 |
| Postcentral gyrus; left | 0.555 |
| Cerebellum exterior; right | 0.553 |
| Calcarine cortex; left | 0.552 |
| Supramarginal gyrus; right | 0.551 |
| Cerebellum white matter; left | 0.551 |
| Cuneus; left | 0.550 |
| Precentral gyrus medial segment; left | 0.547 |
| Posterior cingulate gyrus; left | 0.546 |
| Lingual gyrus; right | 0.545 |
| Postcentral gyrus medial segment; right | 0.542 |
| Cerebellum exterior; left | 0.541 |
| Transverse temporal gyrus; left | 0.539 |
| Calcarine cortex; right | 0.539 |
| Parietal lobe; left | 0.535 |
| Middle cingulate gyrus; right | 0.535 |
| Occipital pole; right | 0.533 |
| Cuneus; right | 0.527 |
| Inferior occipital gyrus; left | 0.525 |
| Parietal lobe; right | 0.525 |
| Cerebellum white matter; right | 0.525 |
| Precuneus; right | 0.525 |
| Middle occipital gyrus; right | 0.523 |
| Frontal pole; right | 0.523 |
| Postcentral gyrus medial segment; left | 0.520 |
| Supramarginal gyrus; left | 0.519 |
| Parietal operculum; right | 0.518 |
| Parietal operculum; left | 0.517 |
| Precuneus; left | 0.514 |
| Posterior cingulate gyrus; right | 0.513 |
| Planum temporale; right | 0.511 |
| Postcentral gyrus; right | 0.510 |
| Superior parietal Lobule; right | 0.509 |
| Vessel; right | 0.509 |
| Occipital lobe; right | 0.509 |
| Occipital pole; left | 0.508 |
| Superior parietal Lobule; left | 0.508 |
| Transverse temporal gyrus; right | 0.507 |
| Frontal pole; left | 0.506 |
| 4th ventricle; total | 0.504 |
| Planum temporale; left | 0.503 |

The performances for the volume of each individual region presented as area under the ROC curve (AUC) for separation of FTD from all other diagnostic groups.

**Table 3A. Performance for all biomarkers with volumes corrected for field strength**

| **ADC+PredictND** | **ALL** | | | **<70 years** | | | **Imaging** |
| --- | --- | --- | --- | --- | --- | --- | --- |
|  | **sens** | **spec** | **AUC** | **sens** | **spec** | **AUC** | **biomarker** |
| FTD vs. non-FTD | 0.58 | 0.94 | 0.85 | 0.62 | 0.95 | 0.89 | API |
| FTD vs. AD | 0.58 | 0.92 | 0.84 | 0.62 | 0.94 | 0.89 | API |
| svPPA+nfvPPA vs. bvFTD | 0.78 | 0.93 | 0.85 | 0.84 | 0.92 | 0.88 | ASI |
| svPPA+nfvPPA vs. bvFTD | 0.69 | 0.8 | 0.76 | 0.73 | 0.78 | 0.77 | TPL |
| svPPA vs. bvFTD | 0.8 | 0.93 | 0.85 | 0.84 | 0.92 | 0.87 | ASI |
| svPPA vs. bvFTD | 0.81 | 0.8 | 0.82 | 0.81 | 0.78 | 0.81 | TPL |

Abbreviations: spec: specificity, sens: Sensitivity, AUC: the area under the receiver operating characteristic curve, FTD: Frontotemporal dementia, Non-FTD: All other diagnostic groups, AD: Alzheimer´s disease, bvFTD: behavioral variant FTD, svPPA: semantic variant primary progressive aphasia, nfvPPA: non-fluent variant primary progressive aphasia, API: the anterior vs. posterior index, TPL: the temporal lobe volume, ASI: the asymmetric index.

**Table 4A. Performance for all biomarkers without 1T images**

| **ADC+PredictND** | **ALL** | | | **<70 years** | | | **Imaging** |
| --- | --- | --- | --- | --- | --- | --- | --- |
|  | **sens** | **spec** | **AUC** | **sens** | **spec** | **AUC** | **biomarker** |
| FTD vs. non-FTD | 0.59 | 0.96 | 0.85 | 0.64 | 0.96 | 0.88 | API |
| FTD vs. AD | 0.59 | 0.94 | 0.85 | 0.64 | 0.95 | 0.89 | API |
| svPPA+nfvPPA vs. bvFTD | 0.81 | 0.91 | 0.84 | 0.83 | 0.89 | 0.85 | ASI |
| svPPA+nfvPPA vs. bvFTD | 0.7 | 0.82 | 0.74 | 0.75 | 0.8 | 0.76 | TPL |
| svPPA vs. bvFTD | 0.82 | 0.91 | 0.85 | 0.81 | 0.89 | 0.84 | ASI |
| svPPA vs. bvFTD | 0.83 | 0.82 | 0.83 | 0.82 | 0.8 | 0.81 | TPL |

Abbreviations: spec: specificity, sens: Sensitivity, AUC: the area under the receiver operating characteristic curve, FTD: Frontotemporal dementia, Non-FTD: All other diagnostic groups, AD: Alzheimer´s disease, bvFTD: behavioral variant FTD, svPPA: semantic variant primary progressive aphasia, nfvPPA: non-fluent variant primary progressive aphasia, API: the anterior vs. posterior index, TPL: the temporal lobe volume, ASI: the asymmetric index.

**Table 5A. Additional baseline characteristics of all cases, the ADC+PredictND cohort**

|  |  |  | | |  | **Non-FTD** | | | | | | |  |
| --- | --- | --- | --- | --- | --- | --- | --- | --- | --- | --- | --- | --- | --- |
|  | **n** | | **FTD**  **n=116** | **Non-FTD**  **N=1098** | | | **AD**  **n=341** | **DLB**  **n=66** | **VaD**  **n=40** | **Other**  **n=104** | **MCI**  **n=229** | **SCD**  **n=317** | **Group wise comparisons**  **when significant** |
| Female, No. (%) | 579 | | 50 (43) | 529 (48) | | | 190 (56) | 10 (15) | 15 (38) | 53 (51) | 88 (38) | 173 (55) | All>DLB. AD,SCD>FTD,VaD,MCI. Other>MCI |
| Age (years) | 1213 | | 64 (7) | 66.8 (9)^†^ | | | 68 (8) | 69 (8) | 71 (8) | 74 (9) | 67 (8) | 62 (9) | Other>AD,DLB,VaD,MCI>FTD,SCD |
| ***Cognitive tests*** |  | |  |  | | |  |  |  |  |  |  |  |
| MMSE | 1213 | | 24 (5) | 25 (4)^†^ | | | 22 (5) | 24 (4) | 24 (4) | 25 (4) | 27 (2) | 29 (1) | SCD,MCI>FTD,DLB,VaD,Other>AD |
| Memory – Learning | 956 | | 27 (9) | 31 (13)^†^ | | | 22 (8) | 25 (9) | 25 (8) | 23 (10) | 31 (9) | 43 (9) | SCD,MCI>All. FTD>AD |
| Memory – Recall | 954 | | 4 (3) | 5 (4)^†^ | | | 2 (2) | 4 (3) | 3 (3) | 3 (3) | 4 (3) | 9 (3) | SCD>All. MCI, FTD, DLB>AD |
| TMT-A, in seconds | 1165 | | 62 (35) | 63 (57) | | | 80 (58) | 103 (69) | 100 (63) | 95 (109) | 48 (20) | 37 (14) | DLB,VaD,Other>FTD>SCD. AD,DLB,VaD,Other>MCI |
| TMT-B, in seconds | 1000 | | 185 (146) | 158 (145) | | | 212 (143) | 295 (207) | 239 (97) | 227 (216) | 146 (136) | 87 (65) | DLB>AD,FTD. AD,DLB,VaD,Other>MCI. All>SCD |
| NPI – Total | 1012 | | 22 (17) | 10 (11)^†^ | | | 12 (11) | 13 (12) | 13 (10) | 14 (16) | 10 (11) | 5 (9) | FTD>All>SCD |
| ***CSF*** |  | |  |  | | |  |  |  |  |  |  |  |
| AB42, pg/ml | 883 | | 881 (291) | 704 (288)^†^ | | | 531 (167) | 730 (258) | 700 (263) | 729 (335) | 751 (310) | 920 (251) | FTD,SCD>MCI,DLB,VaD,Other>AD. |
| Total tau, pg/ml | 870 | | 395 (260) | 486 (344)^†^ | | | 695 (407) | 341 (213) | 305 (160) | 480 (271) | 443 (260) | 290 (166) | AD>All. MCI,Other>SCD |
| P tau, pg/ml | 879 | | 51 (25) | 67 (35)^†^ | | | 86 (39) | 51 (27) | 44 (20) | 65 (31) | 66 (33) | 49 (20) | AD>All. MCI>FTD,DLB,VaD,SCD. Other>SCD |
| ***MRI*** |  | |  |  | | |  |  |  |  |  |  |  |
| API | 1213 | | -2.1 (2) | -0.04 (1)^†^ | | | -0.04 (1) | 0.1 (1) | -0.1 (1) | -0.4 (1) | -0.11 (1) | 0.1 (1) | SCD,DLB>Other. All>FTD |
| ASI | 1213 | | -0.5 (3) | -0.01 (1)^†^ | | | -0.03 (1) | 0.05 (1) | -0.06 (1) | -0.13 (1) | -0.01 (1) | 0.05 (1) | SCD,MCI,AD>FTD |
| Hippocampus, ml | 1213 | | 5 (1) | 6 (1)^†^ | | | 6 (1) | 6 (1) | 6 (1) | 6 (1) | 6 (1) | 7 (1) | SCD>All, MCI>AD,FTD,Other. DLB>AD,FTD, Other |
| Inferior lateral ventricle, ml | 1213 | | 4 (2) | 2 (1)^†^ | | | 3 (1) | 2 (1) | 3 (1) | 3 (2) | 2 (1) | 2 (0) | FTD>AD,VaD,Other>MCI>SCD. VaD,Other>AD>DLB |
| Temporal pole, ml | 1213 | | 10 (3) | 14 (2)^†^ | | | 13 (2) | 14 (2) | 13 (2) | 13 (2) | 14 (2) | 15 (2) | SCD>All>FTD. MCI>AD,Other. DLB>AD |
| Cerebral gray matter, ml | 1213 | | 469 (37) | 502 (34)^†^ | | | 486 (29) | 493 (25) | 479 (33) | 488 (35) | 511 (30) | 520 (33) | SCD>MCI>All. AD,DLB,Other>FTD |
| Cerebral white matter, ml | 1213 | | 381 (34) | 389 (36)^†^ | | | 372 (28) | 385 (32) | 372 (40) | 383 (33) | 390 (33) | 411 (34) | SCD>All. MCI>AD,VaD. DLB,Other>AD |
| Cerebrospinal fluid, ml | 1213 | | 61 (21) | 52 (23)^†^ | | | 60 (20) | 51 (15) | 70 (29) | 63 (31) | 50 (23) | 40 (14) | VaD,Other>DLB. AD,FTD,VaD,Other>MCI. All>SCD |
| Cortical gray matter, ml | 1213 | | 441 (35) | 472 (32)^†^ | | | 457 (28) | 469 (24) | 451 (31) | 459 (33) | 481 (29) | 489 (31) | SCD>All. MCI>AD,FTD,VaD,Other. AD,DLB,Other>FTD |
| Frontal lobe, ml | 1213 | | 173 (18) | 187 (14)^†^ | | | 183 (12) | 187 (11) | 177 (14) | 183 (16) | 189 (13) | 192 (13) | SCD,MCI>AD,FTD,VaD,Other. AD,DLB,Other>FTD. DLB>VaD |
| Temporal lobe, ml | 1213 | | 101 (14) | 115 (10)^†^ | | | 110 (9) | 115 (6) | 113 (10) | 110 (11) | 118 (9) | 121 (8) | SCD>All>FTD. MCI,DLB>AD,Other. |
| Parietal lobe, ml | 1213 | | 100 (9) | 101 (9) | | | 97 (8) | 101 (7) | 96 (8) | 98 (8) | 103 (8) | 105 (8) | SCD>AD,FTD,DLB,VaD,Other. MCI>AD,FTD,VaD,Other. FTD,DLB>AD. DLB>VaD |
| Occipital lobe, ml | 1213 | | 68 (7) | 69 (7) | | | 68 (7) | 67 (6) | 65 (8) | 68 (7) | 70 (6) | 70 (7) | SCD, MCI>AD,DLB,VaD |
| Medial temporal lobe, ml | 1213 | | 15 (3) | 18 (2)^†^ | | | 16 (2) | 18 (2) | 17 (3) | 16 (3) | 18 (2) | 19 (2) | SCD>All>FTD. MCI>AD,Other. Other>DLB>AD |

Abbreviations: FTD: Frontotemporal dementia, AD: Alzheimer´s disease, DLB: Dementia with Lewy bodies, VaD: Vascular dementia, Other: other dementias, MCI: Mild cognitive impairment, SCD: subjective cognitive decline, Memory: the Rey Auditory Verbal Learning Test (RAVLT) values using z-scoring for those with only the Consortium to Establish a Registry for Alzheimer’s Disease (CERAD) word list memory test, TMT: Trail Making Test, NPI: the Neuropsychiatric Inventory, AB42: beta amyloid 1-42, p-tau: tau phosphorylated at threonine 181, API: Anterior vs. posterior index, ASI: the asymmetric index.

Data are presented as mean (SD) or number (%). Differences between groups were assessed using one-way ANOVA with post hoc Bonferroni tests for parametric continuous variables, Kruskal–Wallis for non-parametric variables, and Chi-square test for categorical variables.

^†^ Significant difference between FTD and non-FTD, p<0.05= significant.

**Table 6A. Performance for all biomarkers (API, TPL and ASI), the ADC+PredictND cohort**

a.

| **ADC+PredictND** | **API** | | | | **TPL** | | | | **ASI** | | | | |
| --- | --- | --- | --- | --- | --- | --- | --- | --- | --- | --- | --- | --- | --- |
| **All** | **sens** | **spec** | **AUC** | **sens** | | **spec** | **AUC** | **sens** | | **spec** | **AUC** |  |  |
| FTD vs. non-FTD | 0.59 | 0.95 | 0.83 | 0.69 | | 0.84 | 0.82 | 0.32 | | 0.96 | 0.57 |  |  |
| bvFTD vs. non-FTD | 0.53 | 0.95 | 0.79 | 0.60 | | 0.81 | 0.75 | 0.11 | | 0.94 | 0.46 |  |  |
| svFTD+nfvPPA vs. non-FTD | 0.61 | 0.95 | 0.86 | 0.82 | | 0.95 | 0.91 | 0.76 | | 0.97 | 0.86 |  |  |
| svPPA vs. non-FTD | 0.71 | 0.95 | 0.90 | 0.90 | | 0.95 | 0.95 | 0.80 | | 0.97 | 0.86 |  |  |
| FTD vs. AD | 0.59 | 0.93 | 0.82 | 0.52 | | 0.92 | 0.76 | 0.31 | | 0.91 | 0.57 |  |  |
| bvFTD vs. AD | 0.53 | 0.93 | 0.79 | 0.40 | | 0.91 | 0.67 | 0.07 | | 0.93 | 0.45 |  |  |
| svFTD+nfvPPA vs. AD | 0.61 | 0.93 | 0.86 | 0.82 | | 0.85 | 0.88 | 0.73 | | 0.74 | 0.85 |  |  |
| svPPA vs. AD | 0.71 | 0.93 | 0.90 | 0.91 | | 0.85 | 0.93 | 0.74 | | 0.74 | 0.86 |  |  |
| svPPA+nfvPPA vs. bvFTD | 0.61 | 0.47 | 0.56 | 0.74 | | 0.80 | 0.78 | 0.79 | | 0.92 | 0.85 |  |  |
| svPPA vs. bvFTD | 0.71 | 0.47 | 0.62 | 0.82 | | 0.80 | 0.85 | 0.80 | | 0.92 | 0.85 |  |  |

b.

| **ADC+PredictND** | **API** | | | | **TPL** | | | | **ASI** | | | | |
| --- | --- | --- | --- | --- | --- | --- | --- | --- | --- | --- | --- | --- | --- |
| **<70 years** | **sens** | **spec** | **AUC** | **sens** | | **spec** | **AUC** | **sens** | | **spec** | **AUC** |  |  |
| FTD vs. non-FTD | 0.63 | 0.96 | 0.87 | 0.72 | | 0.85 | 0.85 | 0.32 | | 0.96 | 0.57 |  |  |
| bvFTD vs. non-FTD | 0.58 | 0.96 | 0.85 | 0.65 | | 0.82 | 0.80 | 0.12 | | 0.94 | 0.46 |  |  |
| svFTD+nfvPPA vs. non-FTD | 0.63 | 0.96 | 0.89 | 0.86 | | 0.96 | 0.93 | 0.82 | | 0.97 | 0.90 |  |  |
| svPPA vs. non-FTD | 0.69 | 0.96 | 0.91 | 0.92 | | 0.96 | 0.94 | 0.84 | | 0.97 | 0.89 |  |  |
| FTD vs. AD | 0.63 | 0.95 | 0.88 | 0.55 | | 0.93 | 0.78 | 0.31 | | 0.90 | 0.56 |  |  |
| bvFTD vs. AD | 0.58 | 0.95 | 0.86 | 0.43 | | 0.92 | 0.71 | 0.08 | | 0.92 | 0.45 |  |  |
| svFTD+nfvPPA vs. AD | 0.63 | 0.95 | 0.90 | 0.86 | | 0.85 | 0.90 | 0.76 | | 0.75 | 0.89 |  |  |
| svPPA vs. AD | 0.69 | 0.95 | 0.91 | 0.92 | | 0.85 | 0.93 | 0.76 | | 0.75 | 0.88 |  |  |
| svPPA+nfaPPA vs. bvFTD | 0.63 | 0.42 | 0.55 | 0.76 | | 0.77 | 0.79 | 0.85 | | 0.91 | 0.88 |  |  |
| svPPA vs. bvFTD | 0.69 | 0.42 | 0.58 | 0.82 | | 0.77 | 0.83 | 0.84 | | 0.91 | 0.87 |  |  |

The table presents the sensitivity, specificity and AUC in the ADC+PredictND cohort when addressing the requirement of a specificity around 95 % (cut-off value set at z=-1.65). The results are shown for: a) all subjects and b) subjects <70 years.

Abbreviations: Sens: Sensitivity, spec: specificity, AUC: the area under the receiver operating characteristic curve, FTD: Frontotemporal dementia, Non-FTD: All other diagnostic groups, bvFTD: behavioral variant FTD, svPPA: semantic variant primary progressive aphasia, nfvPPA: non-fluent variant primary progressive aphasia, API: the anterior vs. posterior index, TPL: the temporal lobe volume, ASI: the asymmetric index.

**Table 7A. Performance for all biomarkers (API, TPL and ASI), the DDRC cohort**

a.

| **DDRC** | **API** | | | | **TPL** | | | | **ASI** | | | | |
| --- | --- | --- | --- | --- | --- | --- | --- | --- | --- | --- | --- | --- | --- |
| **All** | **sens** | **spec** | **AUC** | **sens** | | **spec** | **AUC** | **sens** | | **Spec** | **AUC** |  |  |
| FTD vs. non-FTD | 0.58 | 0.94 | 0.85 | 0.67 | | 0.78 | 0.79 | 0.17 | | 0.97 | 0.51 |  |  |
| bvFTD vs. non-FTD | 0.40 | 0.94 | 0.74 | 0.30 | | 0.71 | 0.59 | 0.00 | | 0.97 | 0.36 |  |  |
| svFTD+nfvPPA vs. non-FTD | 0.71 | 0.94 | 0.93 | 0.93 | | 0.94 | 0.94 | 0.29 | | 0.98 | 0.63 |  |  |
| svPPA vs. non-FTD | 0.75 | 0.94 | 0.96 | 1.00 | | 0.94 | 0.99 | 0.33 | | 0.98 | 0.60 |  |  |
| svPPA+nfaPPA vs. bvFTD | 0.71 | 0.60 | 0.67 | 0.79 | | 0.90 | 0.91 | 0.29 | | 1.00 | 0.68 |  |  |
| svPPA vs. bvFTD | 0.75 | 0.60 | 0.69 | 0.83 | | 0.90 | 0.97 | 0.33 | | 1.00 | 0.64 |  |  |

b.

| **DDRC** | **API** | | | | **TPL** | | | | **ASI** | | | | |
| --- | --- | --- | --- | --- | --- | --- | --- | --- | --- | --- | --- | --- | --- |
| **<70 years** | **sens** | **spec** | **AUC** | **sens** | | **spec** | **AUC** | **sens** | | **Spec** | **AUC** |  |  |
| FTD vs. non-FTD | 0.64 | 0.93 | 0.93 | 0.73 | | 0.90 | 0.90 | 0.27 | | 0.97 | 0.61 |  |  |
| bvFTD vs. non-FTD | 0.50 | 0.93 | 0.88 | 0.25 | | 0.80 | 0.71 | 0.00 | | 0.97 | 0.28 |  |  |
| svFTD+nfvPPA vs. non-FTD | 0.71 | 0.93 | 0.96 | 1.00 | | 0.93 | 1.00 | 0.43 | | 1.00 | 0.79 |  |  |
| svPPA vs. non-FTD | 0.67 | 0.93 | 0.95 | 1.00 | | 0.93 | 1.00 | 0.50 | | 1.00 | 0.77 |  |  |
| svPPA+nfaPPA vs. bvFTD | 0.71 | 0.50 | 0.57 | 1.00 | | 1.00 | 1.00 | 0.43 | | 1.00 | 0.86 |  |  |
| svPPA vs. bvFTD | 0.67 | 0.50 | 0.54 | 1.00 | | 1.00 | 1.00 | 0.50 | | 1.00 | 0.83 |  |  |

The table presents the sensitivity, specificity and AUC in the DDRC cohort using the model derived for the ADC+PredictND cohort. The results are shown for: a) all subjects and b) subjects <70 years.

Abbreviations: Sens: Sensitivity, spec: specificity, AUC: the area under the receiver operating characteristic curve, FTD: Frontotemporal dementia, Non-FTD: All other diagnostic groups, bvFTD: behavioral variant FTD, svPPA: semantic variant primary progressive aphasia, nfvPPA: non-fluent variant primary progressive aphasia, API: the anterior vs. posterior index, TPL: the temporal lobe volume, ASI: the asymmetric index.

**Table 8A. Performance at different specificities, the ADC+PredictND cohort**

| **ADC+PredictND** | **spec=0.90** | **spec=0.80** | **spec=0.70** | **optimal** | |  | **Imaging** |
| --- | --- | --- | --- | --- | --- | --- | --- |
|  | **sens** | **sens** | **sens** | **spec** | **sens** | **AUC** | **biomarker** |
| FTD vs. non-FTD | 0.66 | 0.71 | 0.76 | 0.88 | 0.70 | 0.83 | API |
| FTD vs. AD | 0.61 | 0.71 | 0.76 | 0.87 | 0.68 | 0.82 | API |
| FTD vs. SCD | 0.71 | 0.73 | 0.81 | 0.93 | 0.69 | 0.84 | API |
| svPPA+nfvPPA vs. bvFTD | 0.82 | 0.84 | 0.84 | 0.91 | 0.82 | 0.85 | ASI |
| svPPA+nfvPPA vs. bvFTD | 0.55 | 0.71 | 0.76 | 0.78 | 0.76 | 0.78 | TPL |
| svPPA vs. bvFTD | 0.80 | 0.83 | 0.83 | 0.95 | 0.80 | 0.85 | ASI |
| svPPA vs. bvFTD | 0.67 | 0.80 | 0.83 | 0.84 | 0.80 | 0.85 | TPL |

Abbreviations: spec: specificity, optimal = the values that maximize sensitivity + specificity, sens: Sensitivity, AUC: the area under the receiver operating characteristic curve, FTD: Frontotemporal dementia, Non-FTD: All other diagnostic groups, AD: Alzheimer´s disease, SCD: subjective cognitive decline, bvFTD: behavioral variant FTD, svPPA: semantic variant primary progressive aphasia, nfvPPA: non-fluent variant primary progressive aphasia, API: the anterior vs. posterior index, TPL: the temporal lobe volume, ASI: the asymmetric index.

**Table 9A. Baseline characteristics for the BvFTD cases, The ADC+PredictND cohort**

|  |  | | **bvFTD** | |  | |
| --- | --- | --- | --- | --- | --- | --- |
|  | **n** | **API<-1.65** **(n=32)** | | **API>-1.65** **(n=32)** | | **p-value** |
| Female, No. (%) | 65 | 18 (56%) | | 10 (31%)^†^ | | 0.08 |
| Age (years) | 65 | 62 (6) | | 65 (8)^†^ | | 0.04 |
| ***Cognitive tests*** |  |  | |  | |  |
| MMSE – total score | 65 | 24 (4) | | 25 (4) | | 0.51 |
| Memory - learning | 52 | 27 (7) | | 26 (10) | | 0.71 |
| Memory – recall | 51 | 4 (3) | | 4 (3) | | 0.25 |
| TMT-A, in seconds | 60 | 59 (37) | | 60 (26) | | 0.44 |
| TMT-B, in seconds | 51 | 169 (184) | | 238 (125)^†^ | | 0.004 |
| NPI – total score | 55 | 27 (20) | | 21 (12) | | 0.15 |
| ***CSF*** |  |  | |  | |  |
| AB42, pg/ml | 60 | 943 (287) | | 904 (253) | | 0.58 |
| P-Tau, pg/ml | 60 | 53 (33) | | 49 (23) | | 0.69 |
| Total tau, pg/ml | 59 | 450 (405) | | 330 (144) | | 0.32 |
| ***MRI*** |  |  | |  | |  |
| ASI | 65 | 0.6 (3) | | 0.1 (1) | | 0.35 |
| Hippocampus, ml | 65 | 5 (1) | | 6 (1)^†^ | | 0.002 |
| Inferior lateral ventricle, ml | 65 | 4 (2) | | 2 (1)^†^ | | <.0001 |
| Temporal pole, ml | 65 | 9 (2) | | 13 (2)^†^ | | <.0001 |
| Cerebral gray matter, ml | 65 | 456 (38) | | 480 (42)^†^ | | 0.02 |
| Cerebral white matter, ml | 65 | 372 (25) | | 385 (41) | | 0.13 |
| Cerebrospinal fluid, ml | 65 | 69 (24) | | 57 (22) | | 0.05 |
| Cortical gray matter, ml | 65 | 429 (36) | | 452 (39)^†^ | | 0.02 |
| Frontal lobe, ml | 65 | 162 (20) | | 176 (18)^†^ | | 0.003 |
| Temporal lobe, ml | 65 | 99 (12) | | 112 (11)^†^ | | <.0001 |
| Parietal lobe, ml | 65 | 101 (8) | | 98 (9) | | 0.16 |
| Occipital lobe, ml | 65 | 68 (7) | | 66 (7) | | 0.21 |
| Medial temporal lobe, ml | 65 | 14 (3) | | 17 (3)^†^ | | 0.0002 |

Abbreviations: bvFTD: behavioral variant FTD, API: the anterior vs. posterior index, MMSE: the Mini-Mental State Examination, Memory: the Rey Auditory Verbal Learning Test (RAVLT) values using z-scoring for those with only the Consortium to Establish a Registry for Alzheimer’s Disease (CERAD) word list memory test, TMT: Trail Making Test, NPI: the Neuropsychiatric Inventory, AB42: beta amyloid 1-42, p-tau: tau phosphorylated at threonine 181, ASI: the asymmetric index.

Data are presented as mean (SD) or number (%). Differences between groups were assessed using independent *t*-test for parametric continuous variables, Wilcoxon rank sum-test for non-parametric variables, and Chi-square test for categorical variables.

^†^ p<0.05= significant.

**Figure 2A. Relationship between amyloid-β and API in bvFTD cases**

A cut-off value of 675 pg/ml was used as the optimal value to identify AD pathology positivity. A cut-off value z=-1.65 was used for API as the optimal value to identify FTD subjects.

Abbreviations: AB42: Amyloid-β, API: Anterior vs. posterior index
